# Clinical Model for Predicting Warfarin Sensitivity

**DOI:** 10.1038/s41598-019-49329-0

**Published:** 2019-09-06

**Authors:** Zhiyuan Ma, Gang Cheng, Ping Wang, Bahar Khalighi, Koroush Khalighi

**Affiliations:** 1 Easton Cardiovascular Associates, Easton, PA USA; 2 Division of Cardiology, Department of Medicine, Duke University Medical Center, Durham, NC USA; 3 Department of Medicine, Easton Hospital, Easton, PA USA; 4 Division of Surgical Sciences, Department of Surgery, Duke University Medical Center, Durham, NC USA; 5 School of Pharmacy, Temple University, Philadelphia, PA USA; 6 Drexel University College of Medicine, Philadelphia, PA USA; 7 Lehigh Valley Heart Institute, Easton, PA USA

**Keywords:** Cardiovascular biology, Genetics research

## Abstract

Warfarin is a widely used anticoagulant with a narrow therapeutic index and large interpatient variability in the therapeutic dose. Complications from inappropriate warfarin dosing are one of the most common reasons for emergency room visits. Approximately one third of warfarin dose variability results from common genetic variants. Therefore, it is very necessary to recognize warfarin sensitivity in individuals caused by genetic variants. Based on combined polymorphisms in *CYP2C9* and *VKORC1*, we established a clinical classification for warfarin sensitivity. In the International Warfarin Pharmacogenetic Consortium (IWPC) with 5542 patients, we found that 95.1% of the Black in the IWPC cohort were normal warfarin responders, while 74.8% of the Asian were warfarin sensitive (P < 0.001). Moreover, we created a clinical algorithm to predict warfarin sensitivity in individual patients using logistic regression. Compared to a fixed-dose approach, the clinical algorithm provided significantly better performance. In addition, we validated the derived clinical algorithm using the external Easton cohort with 106 chronic warfarin users. The AUC was 0.836 vs. 0.867 for the Easton cohort and the IWPC cohort, respectively. With the use of this algorithm, it is very likely to facilitate patient care regarding warfarin therapy, thereby improving clinical outcomes.

## Introduction

Warfarin is the most widely used oral anticoagulant worldwide. There were more than 25 million prescriptions for warfarin in 2010^1^ and about 7 to 8 million warfarin treatment visits annually between 2009 and 2014 in the United States^2^. Despite its high efficacy, warfarin has a narrow therapeutic window and large interpatient variability with a 10- to 20-fold differences in the therapeutic dose required to achieve target International Normalized Ratio (INR). Because of these challenges associated with warfarin use, it is one of the leading causes in emergency department visits and the most often cited cause of drug-related mortality^3^.

Half of interpatient variability in dose requirement for warfarin could be explained by clinical factors, demographic variables and genetic variants. Of the genetic variants, polymorphisms in cytochrome p450, family 2, subfamily C, polypeptide 9 (*CYP2C9*), and vitamin K epoxide reductase complex, subunit1 (*VKORC1*) independently correlates with warfarin therapeutic dose^4–6^. It is estimated that polymorphisms in *CYP2C9* and *VKORC1* together explain approximately 30% (20–25% for *VKORC1*; 5–10% for *CYP2C9*) of the interpatient warfarin dose variance^4–7^. In view of the strong genetic effects on warfarin dose, the U.S. Food and Drug Administration (FDA) updated the warfarin product label to instruct how to predict individualized dose based on combined polymorphisms of *CYP2C9* and *VKORC1*^8^. Many pharmacogenetic algorithms integrating clinical, demographic and genetic variables have also been developed to predict the dose required in individual patients^9–12^. However, there is a lack of classification for warfarin responses in patients to reflect the genetic influence. In addition, clinical simple tools to identify warfarin sensitivity are needed for clinical use.

In this study, we proposed a classification of warfarin sensitivity based on combined polymorphisms of *CYP2C9* and *VKORC1*. We also developed a clinical algorithm to predict the warfarin sensitivity in patients without laboratory tests for *CYP2C9* and *VKORC1* polymorphisms using a large and diverse data set with patients around the world. We then compared it with a fixed-dose strategy to determine whether the performance of the algorithm was significantly better. Finally, we validated the algorithm externally with an independent cohort to test how generalizable the algorithm is.

## Methods

### The Easton cohort

A total of 492 qualified patients with various cardiovascular diseases were enrolled. Among them, 138 patients were on warfarin therapy. This study protocol complied with the Declaration of Helsinki and was approved by Copernicus Group Institutional Review Boards. Written informed consent was obtained from every patient.

### The international warfarin pharmacogenetic consortium (IWPC) cohort

IWPC Cohort has been reported previously^9,13^. Pooled data containing 6922 chronic warfarin users from four continents were downloaded from the PharmGKB website (http://www.pharmgkb.org/downloads/). This data set records detailed de-identified curated data on demographic factors, clinical features, and genetic variables such as *CYP2C9* and *VKORC1*. Multivariate linear regression was employed to impute missing values for height and weight. Specifically, the height variable was modeled based on weight, race, and sex, while the weight variable was based on height, race, and sex. For missing values of the *VKORC1* rs9923231, the imputation strategy has been described (Table 1)^9^, which is based on linkage disequilibrium in *VKORC1* and race^5^. Imputation of rs9923231 genotype was performed in 13.8% of Asians, 3.6% of blacks, and 20.0% of whites.

**Table 1:** Imputation of *VKORC1* rs9923231 genotype.

**Algorithm**
**If** Race is “Asian” or “White” **and** rs2359612 = ‘C/C’ **then impute** rs9923231 = ‘G/G’
**If** Race is “Asian” or “White” **and** rs2359612 = ‘T/T’ **then impute** rs9923231 = ‘A/A’
**If** Race is “Asian” or “White” **and** rs2359612 = ‘C/T’ **then impute** rs9923231 = ‘A/G’
**If** rs9934438 = ‘C/C’ **then impute** rs9923231 = ‘G/G’
**If** rs9934438 = ‘T/T’ **then impute** rs9923231 = ‘A/A’
**If** rs9934438 = ‘C/T’ **then impute** rs9923231 = ‘A/G’
**If** Race is “Asian” or “White” **and** rs8050894 = ‘G/G’ **then impute** rs9923231 = ‘G/G’
**If** Race is “Asian” or “White” **and** rs8050894 = ‘C/C’ **then impute** rs9923231 = ‘A/A’
**If** Race is “Asian” or “White” **and** rs8050894 = ‘C/G’ **then impute** rs9923231 = ‘A/G’
**Otherwise** keep rs9923231 coded as “Missing”

### Study design and sample

The design and selection criteria for the IWPC Study have been detailed elsewhere^9^. Patients were eligible for the present investigation if they had nonmissing data after imputation on clinical variables, such as warfarin stable dose, height, weight, age, race and use of amiodarone. In addition, we excluded those lacking of *CYP2C9* and *VKORC1* rs9923231 genotypes, low allele frequency of CYP2C9*5, *6, *11, *13 and *14, and an outlier subject with warfarin stable dose 315 mg/week. A total of 5542 subjects were included in this study. The study aimed to develop a clinical algorithm to predict the warfarin sensitivity using clinical variables. The study sample size was based on patients with nonmissing data on clinical and genetic covariates in IWPC and Easton cohorts.

### Warfarin sensitivity genotyping

Genomic DNA was extracted from buccal swab leukocytes using QiangenQiaCube instrument and MagMAX™ DNA Multi-sample kits and concentrated by magnetic bead-based method. *VKORC1* allelic variants rs9923231 (−1639 G > A) in the promoter region were sequenced in 428 patients. CYP2C9*1, *2 and *3 were genotyped in 480 patients.

### Outcome measurement

Warfarin stable dose (mg/week) and incidence of over-anticoagulation event (INR > 5) were used as the major outcome in the Easton cohort. Warfarin stable dose was defined as the dose led to an INR in the therapeutic range (2–3) on at least 3 consecutive INR measurements.

### Warfarin sensitivity

Warfarin sensitivity was defined by the combined profile of *CYP2C9* *1, *2 and *3 and *VKORC1* rs9923231 genotypes based on the FDA warfarin label (Table 2)^8^. *VKORC1* G/G; *CYP2C9* *1/*1, *VKORC1* G/G; *CYP2C9* *1/*2 and *VKORC1* A/G; *CYP2C9* *1/*1 were three compound genotypes for warfarin normal responders. The rest 15 compound genotypes were deemed warfarin sensitive including sensitive and very sensitive groups (Table 2), which requires reduced warfarin dose. Warfarin sensitivity (normal or sensitive) was a categorical variable inputted into binary logistic regression.

**Table 2:** Warfarin sensitivity based on genotypes and three ranges of recommended warfarin doses (mg/week) from the USA FDA drug label (COUMADIN, Reference ID: 3022954).

*VKORC1*	*CYP2C9*					
	*1/*1	*1/*2	*1/*3	*2/*2	*2/*3	*3/*3
G/G	Normal (35–49)	Normal (35–49)	Sensitive (21–28)	Sensitive (21–28)	Sensitive (21–28)	Very Sensitive (3.5–14)
A/G	Normal (35–49)	Sensitive (21–28)	Sensitive (21–28)	Sensitive (21–28)	Very Sensitive (3.5–14)	Very Sensitive (3.5–14)
A/A	Sensitive (21–28)	Sensitive (21–28)	Very Sensitive (3.5–14)	Very Sensitive (3.5–14)	Very Sensitive (3.5–14)	Very Sensitive (3.5–14)

Ranges are derived from multiple published clinical studies. *VKORC1*-1639G > A (rs9923231) variant is used in this table. Other co-inherited *VKORC1* variants may also be important determinants of warfarin dose.

Doses were multiplied by 7 to provide consistent units of milligrams per week.

#### Model selection

Binary logistic regression was used to build a clinical model for predicting warfarin sensitivity. We randomly chose 80% of the eligible patients from IWPC Cohort (stratified according to warfarin sensitivity, for a total of 4433) as the derivation cohort (training data set) to develop logistical regression models. The remaining 20% of the patients (N = 1109) were reserved as the validation cohort (testing data set) to calculate unbiased estimates of correct classification rates. The variables were initially identified based on reported pharmacogenetic dosing algorithm^9^, including warfarin stable dose, height, weight, race, age, enzyme inducer and use of amiodarone. In addition, fixed-dose approaches based on warfarin label were employed to predict warfarin sensitivity, in which warfarin sensitive was inferred when warfarin stable dose was less than intermediate dose between normal and sensitive responders. In this study, both 31 mg/week and 32 mg/week were tested as cutoff values.

#### Cross-Validation and Model Evaluation

The IWPC data set was randomly partitioned into ten equal parts (folds) for a 10-fold cross-validation (CV). Thus, in each iteration, a model was trained on all but one held-aside folds and then tested on the held-aside fold of the data. The iteration was repeated 10 times and each fold served as a test data set to evaluate the model performance. For model quality assessment, the pseudo r-squared measures, the areas under the ROC Curve (AUC), the overall prediction accuracy, the sensitivity and the specificity of the 10-fold CV were evaluated. Logistic regression models were compared with fixed-dose methods using McNemar’s χ^2^ test, in which the numbers of times each model had correct predictions were counted for each patient in a group when a discordant pair occurred between the two models.

### Statistical analysis

For differences in continuous variables, warfarin stable dose, height, and weight between the derivation and validation cohorts (Table 3) were compared with the Wilcoxon rank-sum test. Warfarin doses in whites, blacks and Asians among different sensitivity groups (Table 4) were calculated with the Kruskal-Wallis test. For categorical variables, Fisher exact test was used in *CYP2C9* allele frequencies, χ^2^ tests were used for *VKORC1* rs9923231 genotype, age, and race. Proportions for the use of amiodarone were compared with the z-test. P values < 0.05 were considered to be statistically significant. All analyses were conducted with R (version 3.4.4).

**Table 3:** Demographic and clinical characteristics of the Derivation, Validation and Easton cohorts.

Variable	IWPC Cohort			Easton Cohort (N = 106)
	Derivation Cohort (N = 4433)	Validation Cohort (N = 1109)	P Value^*^	
Warfarin dose—mg/week			0.36	
Median	28.0	28.0		27.5
Interquartile range	20.0–40.1	21.0–41.3		17.5–37.9
Genotype—no. (%)				
*VKORC1* rs9923231			0.34	
G/G	1473 (33.2)	372 (33.5)		35 (33.0)
A/G	1651 (37.2)	389 (35.1)		54 (50.9)
A/A	1309 (29.5)	348 (31.4)		17 (16.0)
*CYP2C9*			0.12	
*1/*1	3330 (75.1)	856 (77.2)		62 (58.5)
*1/*2	587 (13.2)	150 (13.5)		21 (19.8)
*1/*3	403 (9.1)	75 (6.8)		19 (17.9)
*2/*2	47 (1.1)	11 (1.0)		1 (0.1)
*2/*3	51 (1.2)	16 (1.4)		3 (2.8)
*3/*3	15 (0.3)	1 (0.1)		0 (0)
Age—no. (%)			0.65	
<50	738 (16.6)	192 (17.3)		2 (1.9)
50–80	3146 (71.0)	790 (71.2)		56 (52.8)
>80	549 (12.4)	127 (11.5)		48 (45.3)
Height—m			0.35	
Median	167.6	167.6		170.2
Interquartile range	160.0–175.4	160.0–176.5		162.6–175.3
Weight—kg				
Median	75.9	76.0	0.76	80.5
Interquartile range	63.0–90.7	63.0–91.0		69.9–94.3
Race—no. (%)			0.44	
White	2418 (54.5)	585 (52.8)		106 (100)
Asian	1161 (26.2)	298 (26.9)		
Black	492 (11.1)	140 (12.6)		
Mixed or missing	362 (8.2)	86 (7.8)		
Amiodarone	211 (4.8)	58 (5.2)	0.51	10 (9.4)

*P values for the difference between the derivation and validation cohorts were calculated with the use of the Wilcoxon rank-sum test for warfarin dose, height, and weight, Fisher’s exact test for *CYP2C9* genotype, χ^2^ tests were used for *VKORC1* rs9923231 genotype, age, and race, and the z-test for proportions for the use of amiodarone.

**Table 4:** Warfarin sensitivity in the IWPC and Easton cohorts.

Warfarin Sensitivity Dose—mg/week	IWPC Cohort					Easton Cohort
	All (N = 5542)	White (N = 3003)	Black (N = 632)	Asian (N = 1459)	P Value	White (N = 106)
Normal						
Median	35.0	36.0	40.0	28.0	<0.001^*^	35.0
Interquartile range	28.0–47.5	28.0–48.0	30.0–52.5	21.0–38.5		25.0–44.4
Prevalence—n. (%)	3059 (55.2)	1861 (62.0)	601 (95.1)	288 (19.7)	<0.001^#^	59 (55.7)
Sensitive						
Median	21.0	25.0	35.0	21.0	<0.001^*^	18.5
Interquartile range	17.5–28.0	18.0–32.7	27.5–45.0	14.0–26.8		14.9–32.5
Prevalence—n. (%)	2285 (41.2)	1038 (34.6)	29 (4.6)	1091 (74.8)		40 (37.7)
Very Sensitive						
Median	14.0	14.0	13.7	14.0	0.93^*^	12.0
Interquartile range	10.5–19.3	10.9–20.0	11.5–15.8	10.5–17.9		11.1–14.0
Prevalence—n. (%)	198 (3.6)	104 (3.5)	2 (0.3)	80 (5.5)		7 (6.6)

*P values for warfarin dose for each race in different sensitivity groups were calculated with the use of the Kruskal-Wallis test. ^#^P value was calculated with the use of the Fisher’s exact test.

## Results

### Basic characteristics of the study cohorts

The characteristics of the patients are shown in Table 3. In the Easton cohort, of 138 patients on long-term warfarin therapy for thromboembolic disorders and other cardiovascular diseases, 106 patients were included for analyses with complete clinical and genotype data. All patients reached the therapeutic warfarin stable dose, defined as the dose of warfarin required to maintain an INR between 2 and 3. The median warfarin stable dose was 27.5 mg/week. The percentages of patient age less than 50, 50 to 80, and older than 80 were 1.9%, 52.8%, and 45.3%, respectively. Among them, 9.4% (10/106) of patients were concomitantly taking amiodarone.

There were 5542 patients with a median warfarin stable dose of 28.0 mg/week included for analyses in the IWPC cohort. Th INR for all patients fell within the target range of 1.7 to 3.3, with the majority maintained between 2 and 3. The proportions of patient age in the IWPC cohort less than 50, 50 to 80, and older than 80 were 16.8%, 71.0%, and 12.2%, respectively. The percentage of patients concomitantly taking amiodarone was 4.9% (269/5542).

### Warfarin sensitivity profiles of the study cohorts

**Figure 1:**
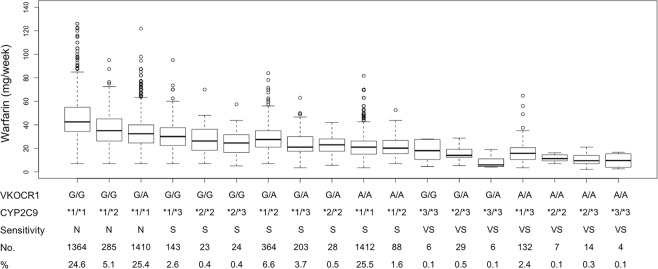
Warfarin stable dose across different genotypes. N: normal; S: sensitive; VS: very sensitive.

To better profile the warfarin responses in different individuals, we classified the warfarin sensitivity into 3 categories (normal, sensitive and very sensitive) base on combined polymorphisms in *CYP2C9* and *VKORC1* in the FDA warfarin label (Table 2). Warfarin stable doses for different warfarin sensitive groups in the IWPC cohort are shown in Fig. 1. There were 44.8% of warfarin sensitive patients overall. In the normal group, *VKOCR1* G/G; *CYP2C9* *1/*1 (24.6%) and *VKORC1* A/G; *CYP2C9* *1/*1 (25.4%) were the two most common genotypes, while *VKOCR1* G/A; *CYP2C9* *1/*2 (6.6%) and *VKORC1* A/A; *CYP2C9* *1/*1 (25.5%) in the sensitive group and *VKOCR1* G/A; *CYP2C9* *2/*3 (0.5%) and *VKORC1* A/A; *CYP2C9* *1/*3 (2.4%) in the very sensitive group were the two most common genotypes, respectively. As shown in Table 4, the median warfarin stable doses (40 mg/week and 35 mg/week) for the Black in the normal and sensitive group were higher than those (36 mg/week and 25 mg/week) in White (P < 0.001), whereas the median warfarin stable doses (28 mg/week and 21 mg/week) for the Asian in the normal and sensitive group were the least among the three races (P < 0.001). In line with this, 95.1% of the Black in the IWPC cohort were normal warfarin responders, in contrast to 74.8% of the Asian being warfarin sensitive (P < 0.001), indicating race is a contributing factor for warfarin sensitivity. In the Easton cohort, the median warfarin stable dose for the normal, sensitive and very sensitive groups were 35.0 mg/week, 18.5 mg/week, and 12.0 mg/week, respectively.

### Incidence of INR >5

It has been shown that the intensity of anticoagulant therapy is the most important risk factor for bleeding, which is the main complication of oral anticoagulant therapy and that hemorrhagic events increase exponentially as the INR increases >5.0^14^. Therefore, the incidence of INR measurements >5 is often used as a surrogate marker for bleeding complications during warfarin therapy^3^. As shown in Table 5, the average incidence of INR >5 in the sensitive and very sensitive combined group was 1.02, which was nearly 2-fold more frequent than that (0.36) in the normal group in the Easton cohort (P = 0.03), although the median follow-up time was 44 months in the sensitive and very sensitive group compared with 32 months in the normal group (P = 0.05), suggesting warfarin sensitive patients are more prone to bleeding complications.

**Table 5:** Times of over-anticoagulation events in the Easton cohort.

	Normal	Sensitive + Very Sensitive	P Value^*^
INR >5	0.36 ± 0.12	1.02 ± 0.27	0.03
Follow-up (months)			0.05
Median	32	44	
Interquartile range	6–42	24–66	

*P values were calculated with the use of the unpaired two-sided t test.

### Comparison of the prediction models

To develop a parsimonious model to predict warfarin sensitivity, stepwise logistic regression was used to identify the important features. In logistic regression analyses, the variables warfarin stable dose, height, weight, race, age, and use of amiodarone were found to be significantly associated with warfarin sensitivity. Moreover, fixed-dose approaches based on the FDA warfarin label were also tested for clinical utility. The performance of the logistic regression and fixed-dose models in the derivation and validation cohorts is shown in Table 6. The logistic regression model provided significantly better prediction than the fixed-dose approach. With a threshold of probability >0.4 for warfarin sensitivity, the accuracy, sensitivity, and specificity in the logistic regression were 0.781, 0.824, and 0.746 for the derivation cohort and 0.795, 0.831, and 0.766 for the validation cohort, while those using the fixed-dose method with a cutoff value of warfarin stable dose less than 31 mg/week were 0.717, 0.799, and 0.651 for the derivation cohort and 0.743, 0.819, and 0.676 for the validation cohort (P < 0.001), respectively. Similarly, the accuracy, sensitivity, and specificity in the logistic regression with a threshold of probability (P > 0.35) were 0.769, 0.862, and 0.693 for the derivation cohort and 0.784, 0.863, and 0.721 for the validation cohort, while those using the fixed-dose method with a cutoff value of warfarin stable dose less than 32 mg/week were 0.702, 0.837, and 0.593 for the derivation cohort and 0.743, 0.845, and 0.660 for the validation cohort (P = 0.038), respectively. The AUC was used as an additional metrics for evaluating the logistic regression model. The closer AUC is to 1, the greater is the predictive ability of the model. In the logistic regression model, the AUC was 0.865 for the derivation cohort and 0.878 for the validation cohort. Moreover, the model performance was evaluated by 10-fold CV. The logistic regression model exhibited a consistent predictive power with an AUC of 0.867 ± 0.014 (Table 6), indicating the strong robustness of the model. To investigate the importance of selected features in the clinical prediction model, the AUC were determined after excluding each variable in the model. The AUC without warfarin stable dose or race by CV were 0.766 ± 0.109 and 0.823 ± 0.025, respectively, indicating that warfarin stable dose plays a crucial role in the model.

**Table 6:** Model performance between the clinical algorithm and fixed-dose approach.

Prediction Model	AUC	Accuracy	Sensitivity	Specificity	Accuracy	Sensitivity	Specificity
Clinical algorithm		Cutoff (P > 0.40)			Cutoff (P > 0.35)		
Derivation Cohort	0.865	0.781	0.824	0.746	0.769	0.862	0.693
Validation Cohort	0.878	0.795	0.831	0.766	0.784	0.863	0.721
10-fold CV (Mean ± SD)	0.867 ± 0.014	0.782 ± 0.019	0.825 ± 0.025	0.747 ± 0.026	0.770 ± 0.018	0.862 ± 0.027	0.694 ± 0.027
Fixed-dose		Warfarin dose (mg/week) <31			Warfarin dose (mg/week) <32		
Derivation Cohort		0.717	0.799	0.651	0.702	0.837	0.593
Validation Cohort		0.743	0.819	0.676	0.743	0.845	0.660
10-fold CV (Mean ± SD)		0.722 ± 0.013	0.803 ± 0.030	0.656 ± 0.016	0.721 ± 0.014	0.827 ± 0.030	0.635 ± 0.019
P Value			<0.001^*^			0.038^#^	

*P value for the differences between clinical algorithm with a threshold of probability >0.4 for warfarin sensitivity and the fixed-dose method with a cutoff value of warfarin stable dose less than 31 mg/week was calculated with the use of the McNemar’s test. ^#^P value for the differences between clinical algorithm with a threshold of probability >0.35 for warfarin sensitivity and the fixed-dose method with a cutoff value of warfarin stable dose less than 32 mg/week.

### Final model and external validation

**Figure 2:**
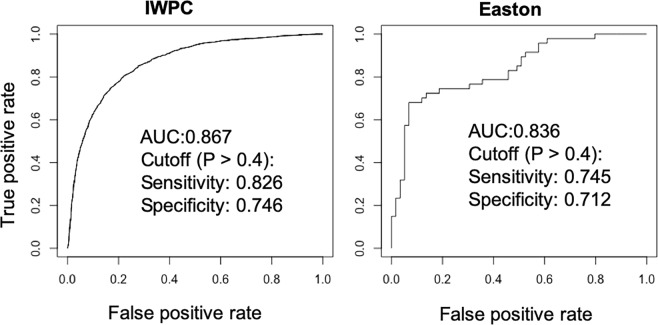
ROC curve analysis of the IWPC and Easton cohorts using the warfarin sensitivity algorithm.

To fully utilize the IWPC data set, we pooled the derivation and validation cohorts and rederived a final logistic regression model using the same variables. The odds ratios and pseudo r-squared measures for regression are shown in Table 7. The clinical refinement algorithm is shown in Table 8. We next tested the final logistic regression model using the external Easton cohort (Fig. 2). The AUC was 0.836 for the Easton cohort and 0.867 for the whole IWPC cohort. With the threshold of probability > 0.4 for warfarin sensitive, the sensitivity and specificity were 0.745 and 0.712 for the Easton cohort, 0.826 and 0.746 for the IWPC cohort, respectively.

**Table 7:** Regression Odds Ratios and Pseudo R-squared Measures.

Variables	Odds ratio	95% CI	P Value
Square root of weekly warfarin stable dose	0.336	(0.312–0.363)	<0.001
Height in cm	1.008	(0.999–1.016)	0.075
Weight in kg	1.012	(1.007–1.016)	<0.001
Age in decades	0.764	(0.726–0.804)	<0.001
Black or African American	0.020	(0.013–0.031)	<0.001
White	0.242	(0.199–0.293)	<0.001
Missing or Mixed race	0.141	(0.108–0.185)	<0.001
Amiodarone status	0.502	(0.369–0.683)	<0.001
Pseudo r-squared measures	Cox and Snell 0.368	Nagelkerke 0.492	McFadden 0.333

**Table 8:** Logistic regression model for warfarin sensitivity.

**Warfarin sensitivity algorithm**		
	6.3606	
−	1.0903 ×	Square root of weekly warfarin stable dose
+	0.0075 ×	Height in cm
+	0.0116 ×	Weight in kg
−	0.2693 ×	Age in decades
−	3.8913 ×	Black or African American
−	1.4203 ×	White
−	1.9562 ×	Missing or Mixed race
−	0.6882 ×	Amiodarone status
=	**Z (log odds)**	
**Probability (P)** = **1** − **1/(1** + **exp(Z)); If P** > **0**.**4**, **warfarin sensitive**.		

Black or African American = 1 if race is Black, otherwise 0; White = 1 if race is White, otherwise 0; Missing or mixed race = 1 if race is unspecified or mixed, otherwise 0; amiodarone = 1 if patient taking amiodarone, otherwise 0.

## Discussion

Due to a narrow therapeutic index and interpatient variability, it is of great importance to recognize the warfarin response individually. It has been shown in warfarin pharmacogenetics that genetic variants in *CYP2C9* and *VKORC1* explain approximately one third of the interpatient dose variance^4–6^ and are more strongly associated with warfarin stable dose than all other known patient factors. Given a strong genetic basis underlying warfarin stable dose, here we proposed a simple clinical classification for warfarin response in individual patients based on the FDA warfarin label to better represent and recapitulate the genetic variants. To better understand the distribution of warfarin sensitivity across different races, we examined the IWPC cohort and found that 95.1% of the Black were normal warfarin responders, while 74.8% of the Asian were warfarin sensitive. This discrepancy is attributing to minor allele frequencies of *VKORC1* SNP rs9923231 and *CYP2C9* SNP rs1799853 (*2) and rs1057910 (*3) between ethnicities, with more genetic variation among individuals of European descent than in individuals of African and Asian descent^13^. In addition, although the majority of the Black in IWPC cohort was normal warfarin responders, it is worth noting that rare *CYP2C9* variant alleles (CYP2C9*5, *6, *8, and *11) with reduced enzymatic activity contribute to dose variability among African Americans^15,16^.

Bleeding is the main complication during warfarin therapy, which is well correlated with the incidence of INR measurements >5. Homozygosity for the *VKORC1* −1639 G > A (A/A) has been reported to be associated with a significantly increased number of INR >5 and occurrence of bleeding events during the first month of therapy, compared to the G/G genotype^17^. Consistent with this finding, we found that the average incidence of INR >5 in the sensitive and very sensitive combined group was 1.02, compared to 0.36 in the normal group in the Easton cohort (P = 0.03). Moreover, it has been reported that sensitive warfarin responders require pharmacogenomic-guided protocols to achieve well-controlled INR, while normal warfarin responders only need a fixed-dose or clinical protocol to achieve well-controlled INR^18^. Taken together, these data suggest warfarin sensitive responders are predisposed to out-of-range INR than warfarin normal responders. Therefore, it is reasonable to frequently monitor INR in warfarin sensitive patients.

Since warfarin sensitivity contributes to adverse events including bleeding, recognizing warfarin sensitivity is crucial to patient care. In this study, we developed a clinical model to predict warfarin sensitivity. We foresaw how it could be utilized in several clinical scenarios. First, patients who are at increased risk of bleeding complications, such as history of gastrointestinal tract bleeding or patients who suffer bleeding events while on warfarin therapy. In the case of bleeding, our algorithm may help to determine whether the underlying cause of bleeding was due to warfarin sensitivity as a result of genetic variants. Second, patients who schedule for warfarin withdrawal prior to an invasive procedure. It has been reported that patients with two *CYP2C9* variant alleles (CYP2C9*2/*2 or CYP2C9*2/*3), the odds of having an INR of ≥1.5 before the planned day of surgery is 8.64 times greater (95% confidence interval 2.25–33.25) than for other patients^19^. This algorithm could facilitate the patient management by reducing potential harm resulting from either discontinuing warfarin too early (thus predisposing the patient to thrombosis) or stopping it too late (thus at increased risk of perioperative bleeding). In general, if patients with predicted warfarin sensitive, we recommend increasing the frequency to monitor INR compared to those with normal warfarin response and determining genetic variants by warfarin sensitivity tests. Of note, personized genotype-guided warfarin dosing has demonstrated clinical benefits and superior clinical outcomes in major clinical trials^20–22^, future studies will investigate how warfarin sensitivity algorithm can be used along with pharmacogenetic dosing algorithms.

The clinical model we developed includes 6 variables, such as warfarin stable dose and race. The importance of selected features in the prediction model was determined after excluding each variable by the relative AUC changes. The warfarin stable dose was the most important variable to predict warfarin sensitivity. Moreover, compared to the fixed-dose strategy, i.e. warfarin stable dose, the clinical model provided significantly better prediction, especially for specificity. From the clinical point of view, many patients with warfarin stable doses, but unknown of warfarin sensitivity as discussed above in several clinical scenarios could be predicted more accurately by using the clinical model than just by warfarin stable doses, thereby improving clinical outcomes.

Several limitations are present in our study. First, missing genotypes of *VKORC1* in the IWPC cohort for some patients were imputed based on linkage disequilibrium^5^. Missing values for height and weight were imputed using multivariate linear regression. Although these imputation strategies are generally reliable, some errors could have been introduced in the study. Second, as shown in Fig. 1, warfarin resistance (>70 mg/week) existed in many patients in the IWPC cohort, especially in warfarin normal groups. The polymorphisms in *VKORC1* and *CYP2C9* associated with warfarin resistance were not explored^23,24^. Future studies need to identify additional genetic variants to differentiate warfarin normal and resistant patients. Third, additional potential variables affecting the prediction of warfarin sensitivity were not included in the model, such as comorbidities, additional drug–drug interactions, and patient behaviors, including diet, exercise, and compliance. Future studies are needed to determine how important these additional potential variables are for the prediction of warfarin sensitivity. Of note, with more variables integrated into the model, better performance is achieved at the risk of overfitting and reduced model simplicity.

In conclusion, according to the FDA warfarin label based on genetic variants in *CYP2C9* and *VKORC1*, we established a clinical classification for warfarin sensitivity. In our Easton cohort, warfarin sensitive caused an increased incidence of out-of-range INR in patients than that of warfarin normal responders. In addition, using data from a large and diverse cohort of patients from IWPC, we developed and validated a clinical algorithm to predict warfarin sensitivity in individual patients. This clinical algorithm performed better than a fixed-dose approach. With the use of this algorithm, it is very likely to facilitate patient care associated with warfarin therapy in a number of clinical scenarios, thereby improving clinical outcomes.

## Data Availability

The IWPC data set was downloaded from the PharmGKB website. The Easton data set analyzed during the current study is available from the corresponding author on reasonable request.
